# NIA Division of Neuroscience

**DOI:** 10.1093/geroni/igaf122.1558

**Published:** 2025-12-31

**Authors:** Jennie Larkin

**Affiliations:** National Institute on Aging, Bethesda, Maryland, United States

## Abstract

Dr. Larkin will discuss research foci of the NIA Division of Neuroscience. Dr. Larkin will also be available for small group discussion.

